# A multidimensional implicit approach to gender stereotypes

**DOI:** 10.3389/fpsyg.2023.1280207

**Published:** 2023-11-10

**Authors:** Sara Panerati, Monica Rubini, Valeria A. Giannella, Michela Menegatti, Silvia Moscatelli

**Affiliations:** ^1^Department of Psychology ‘Renzo Canestrari’, Alma Mater Studiorum, University of Bologna, Bologna, Italy; ^2^Department of Psychology, The Catholic University of the Sacred Heart, Milan, Italy

**Keywords:** gender inequalities, implicit method, competence, dominance, sociability, morality, attractiveness

## Abstract

Research has widely explained gender inequalities in terms of gender stereotypes, according to which women are considered more nurturing, empathic, and emotional but less competent – than men. Recent evidence highlights that especially women are portrayed along multiple dimensions. In this research, we adopted an implicit Semantic Misattribution procedure to detect whether gender stereotypes have a multidimensional structure and are differently attributed to men and women. Results showed that Competence and Dominance-related terms were considered more masculine ones. In contrast, Morality and Physical Attractiveness were attributed to feminine ideograms to a higher and significant extent than masculine ones. Sociability was related to feminine and masculine ideograms almost to the same extent. The gathered evidence provided a multidimensional picture even composed of more judgment dimensions with reference to women highlighting how it can be difficult for them to meet all those multiple expectancies.

## 1. Introduction

Among the causes of gender inequalities, social psychological research has consistently documented the role of gender stereotypes that, initially have been conceived to be organized along two dimensions referring to goals and relations and being labeled as competence and warmth (Fiske et al., 2002), communion and agency (Abele et al., 2016), and competence and morality (Wojciszke, 2005), respectively. Along this line, Fiske et al. (2002) have shown that men are usually depicted as competent (e.g., intelligent, confident, competitive, and independent) but not very nice (e.g., sincere, warm, and tolerant). In contrast, women are seen as nice but not very competent. In more specific terms, the agency dimension refers to the ability to be performative and goal-oriented. It involves qualities such as efficiency, intelligence, strength, and capability, while the communion dimension pertains to benevolence in social relations and involves qualities such as friendliness, kindness, cooperativeness, and trustworthiness (Abele et al., 2008). Not adhering to gender expectancies usually leads to adverse outcomes and penalties, such as those related to the shifting standard effect (Biernat, 2009), according to which women and men are evaluated by setting different standards in personnel evaluation. This usually leads to setting lower minimum standards for women in the initial screening phase of recruitment procedures. However, higher confirmatory standards are required for women than men (Biernat and Fuegen, 2001). Moreover, backlash effects (Rudman and Glick, 2001) may emerge as women who display competence attributes (e.g., demonstrating self-assertion and achievement orientation) can represent a violation of gender prescriptions and produce social disapproval and negativity, leading to a decreased likelihood of being hired (Cortina et al., 2021) and lower promotion opportunities (Rudman and Phelan, 2008).

Going beyond a bi-dimensional approach, it has been contended that the warmth or communion dimension encompasses two distinct components referring to morality and sociability, given the fact that individuals can be sociable without being moral/honest, or they can be moral/honest without being sociable (e.g., Leach et al., 2007; Brambilla and Leach, 2014; Abele et al., 2016). Along this line, scholars have disentangled the components of agency from competence as a distinct factor (e.g., Carrier et al., 2014) and have subdivided the agency dimension into several characteristics, such as self-reliance and dominance (Schaumberg and Flynn, 2017), assertiveness, competence, and effort (Louvet et al., 2019). Moreover Hentschel et al. (2019) explored intra-dimension characteristics of agency and communion: assertiveness, independence, instrumental competence, leadership competence (agency dimension), and concern for others, sociability and emotional sensitivity (communality dimension). Results indicated that stereotypes about communality persist and were equally prevalent for male and female participants, but agency characterizations were more complex. Male participants generally described women as being less agentic than men. Female participants differentiated among agency characteristics and described women as less assertive than men but as equally independent and leadership competent. Both male and female participants considered men and women equally high on instrumental competence.

### 1.1. A multidimensional framework of gender stereotypes

Following this line of thought, some studies investigated whether, when addressing gender stereotyping phenomena, it is more realistic to adopt a multidimensional framework (e.g., Abele et al., 2016; Hentschel et al., 2019). Prati et al. (2019) examined gender inequality in personnel selection by considering competence, sociability, and morality, by analyzing spontaneous reference to characteristics considered to be owned by men and women in a performance appraisal procedure within the public administration field. The evaluation reports of professional selectors showed that women’s assessment relies on multiple bases: women need to fulfill more expectancies than men, whereas men are evaluated based primarily on their competence. In other words, individuals rely on more complex requirements when evaluating women rather than men. Moscatelli et al. (2020) confirmed and extended these findings by examining the relative importance of competence, morality, and sociability in employment decisions by content-analyzing archival reports of professionals and by investigating the importance of different characteristics in hiring a female or male candidate for a job position. Findings consistently showed that competence was the most crucial dimension in the evaluations and decisions concerning male candidates, whereas all dimensions were important for female candidates. This tendency has been labeled Perfection Bias (Moscatelli et al., 2020) since multiple criteria influence decisions concerning women, and consequently, women are requested to satisfy more requirements than men, thus expectancies of “perfection.” Similar expectations of perfection are reflected in several aspects of their working life, for instance, the hiring process (Brescoll, 2016) and career progression (Tabassum and Nayak, 2021).

These expectations of perfection toward women also influence the formation of selectors’ first impressions through candidates’ pictures. Menegatti et al. (2021) considered how candidates’ competence, morality, sociability, and attractiveness inferred from the candidate’s face influenced hiring decisions for men and women. Findings revealed that female candidates’ facial competence predicted the hiring decision. Moreover, the selection of female candidates relied also on morality and attractiveness inferred from their faces. In this regard, it could be argued that attractiveness constitutes a relatively irrelevant characteristic in job recruitment unless job selection concerns, for example, a fashion model. Nevertheless, findings showed that it constitutes a social judgment criterium influencing discrimination (e.g., Axt et al., 2019). Accordingly, attractive individuals receive advantageous treatments in various life domains, including work (Jawahar and Mattsson, 2005; Zebrowitz, 2017). Extending this multidimensional approach, Pireddu et al. (2022) investigated the impact of gender stereotypes on perceived leadership suitability of women and men. In addition to the characteristics considered in the perfection bias studies (i.e., competence, morality, sociability, and attractiveness), dominance was also investigated since it is strongly associated with leadership stereotypes (Bongiorno et al., 2021). Moreover, women are considered to perform negatively on dominance (Williams and Tiedens, 2016). The evidence of Pireddu et al. (2022) highlighted that attractiveness and competence were the most important predictors of hiring likelihood for all candidates. Moreover, morality and sociability were more critical in evaluating men than women, while dominance was rated as more important in evaluating women than men. The authors concluded that these findings suggested an evolution of gender expectancies since counter-stereotypical characteristics of male and female candidates received more weight in assessing the candidates.

### 1.2. Implicit measures of gender stereotypes

Most studies on gender stereotypes have employed explicit methods, whereas few have addressed the issues by adopting implicit methods (White and White, 2006). Thus, new ways of investigation can be helpful to shed light on more subtle ways through which gender stereotypes are vehiculated (Bhatia and Bhatia, 2021). Since recent findings suggest that multidimensional judgments affect women’s evaluations, would it be possible to detect this tendency also at an implicit level? Several studies demonstrated that gender stereotypes are usually activated automatically (i.e., Lai and Wilson, 2021) and, therefore, barely controlled (Moors and De Houwer, 2006). Therefore, some studies pointed out the inconsistency between the results obtained through implicit and explicit measures since the lower level of stereotypes emerges from self-report studies. For instance, Nosek et al. (2007) showed that stereotypes are pervasive while corresponding self-report measures exhibit substantially lower rates of prejudice and stereotypes. Such evidence has given rise to the conviction that it could be beneficial to investigate the phenomenon by implementing implicit measures since they are less susceptible to self-presentation concerns.

When people are requested to provide a judgment on a specific topic, they can have an implicit reaction but may restrain themselves from expressing it (Nosek et al., 2011). Hence, one of the most common methods employed is the Implicit Association Test (i.e., IAT; Greenwald et al., 1998), which investigates the association’s strength between two elements by considering the time reactions of the participants. This type of task has been primarily adopted to study gender stereotypes. For instance, studies pointed out a backlash effect against agentic women (Rudman and Glick, 2001) and a gendered evaluation of roles such as engineer as a masculine one and teacher as a feminine one (White and White, 2006), thus showing a stronger association between science and men than science and women (Nosek et al., 2011). Moreover, implicit methods have been showing exciting results investigating, among others, how stereotypes beyond people’s awareness affect women’s career progression (Teelken et al., 2021), the evaluations regarding the stereotypical perception of the type of occupations (i.e., engineer, accountant, and the teacher) and their evaluations in terms of masculinity vs. femininity (White and White, 2006), and the associations between gender and liberal art vs. science (Rezaei, 2011).

Besides the measures based on time reaction, it has been proved that participants’ responses are susceptible to the influence of the priming procedure (e.g., Gawronski and Bodenhausen, 2006). For instance, Rudman and Phelan (2008) investigated the priming effects on women’s leadership self-concept. The procedure consisted of two prime conditions: the traditional one depicted men as occupying traditional roles (e.g., Stanford business professor, business executive), while the non-traditional priming provided opposite associations of women with traditional male roles. Findings showed that women in the traditional priming condition displayed higher automatic gender stereotypes, leading to a decreasing interest in masculine jobs. Thus, these methods are based on the idea that our minds constantly create associations among concepts and feelings (Cameron et al., 2012). Among the priming methods, one of the most applied to the study of gender stereotypes is represented by the Affective Misattribution Procedure (i.e., AMP), which is designed to assess spontaneous behavior arising from the activations of affective states (e.g., Imhoff et al., 2011). Generally, the AMP is composed of several trials in which ambiguous prime stimuli (e.g., a positive vs. negative image) are presented several times to the participants, each of them followed by a Chinese ideogram (for a review, see Payne and Lundberg, 2014). Then, participants are requested to evaluate the ideogram regarding agreeability (e.g., pleasant vs. unpleasant). Therefore, this procedure focuses on participants’ spontaneous affective answers to the first (ambiguous) stimulus, which is erroneously considered due to the second stimulus (e.g., Mann et al., 2019).

This misattribution process has been implemented not only to observe associations on an affective level but also on a semantic one. The Semantic Misattribution Procedure (i.e., SMP) represents a variant of the AMP to investigate implicit associations focusing on spontaneous behavior related to activating semantic concepts.

Despite being relatively recent, this procedure was very versatile for the study in various fields of social psychology, such as gender stereotypes, (for a review, see Vezzoli and Zogmaister, 2016) and possesses good psychometric properties (Ye and Gawronski, 2018). Gawronski and Ye (2014) investigated whether stereotypical male or female roles would be implicitly associated with men or women (e.g., doctor-male; nurse-women). Participants’ trials consisted of stereotypical working positions, as prime, followed by a Chinese ideogram. Findings showed that participants usually evaluated as feminine the ideographs after being primes with a feminine stereotypical role, while the opposite occurred in front of male prime words. Few more studies (Ye and Gawronski, 2018) have implemented this type of procedure to investigate gender stereotypes, showing how this tool has significant advantages in advancing knowledge on the topic that explicit methods would not entirely capture.

### 1.3. The current research

To fill this gap, based on the studies that adopted a multidimensional approach to gender stereotypes (e.g., Hentschel et al., 2019; Prati et al., 2019; Moscatelli et al., 2020; Menegatti et al., 2021; Pireddu et al., 2022), the goal of this study was to address the possible multidimensional nature of gender stereotypes at an implicit level. Therefore, we investigated the implicit semantic associations of competence, morality, sociability, dominance, and attractiveness with ideograms that refer to masculine or feminine words. In light of previous literature, we hypothesize that traits traditionally aligned with masculinity, namely Competence (Fiske et al., 2002) and Dominance (Bongiorno et al., 2021; Pireddu et al., 2022), will show a stronger association with masculine ideograms than traits like Morality (Prati et al., 2019; Moscatelli et al., 2020), Sociability, and Attractiveness (Hosoda et al., 2003; Menegatti et al., 2021). In essence, we expect Morality, Sociability, and Attractiveness to be less frequently linked with Masculinity in comparison to Competence and Dominance.

Furthermore, we aimed to explore differences in implicit attributions made by male and female participants since as reviewed above Hentschel et al. (2019) found discrepancies in how men and women portray their gender.

## 2. Material and method

An *a priori* power analysis was conducted using G*Power version 3.1.9.7 (Faul et al., 2007) to determine the minimum sample size required to test the study hypothesis. Results indicated the required sample size to achieve 90% power for detecting a medium effect, at a significance criterion of *α* = 0.05, was *N* = 104 for a repeated-measure ANOVA. Thus, the obtained sample size of *N* = 108 is adequate to test the study hypotheses.

### 2.1. Participants

One hundred and eight (69 women, 38 men, 1 not specified, *M*_age_ = 24.53, *SD*_age_ = 8.14) students took part in the study. Since the stimuli of the study were presented in English, we evaluated their proficiency in English by asking them to translate into Italian a battery of English words and sentences. Thirteen students were excluded for not having sufficient English mastery. Moreover, all participants reported not having any mastery of Chinese. Most of the participants had completed secondary education (57.4%), followed by those who held a bachelor’s degree (26.9%), a master’s degree (13%), and a doctoral degree (1.9%). Almost all participants were native Italian speakers (98%). Participants were all Italian except for one with American nationality and one with Italian-Albanian nationality.

### 2.2. Procedure

Ethics approval was obtained by the Bioethical Committee of the University (blinded) in November 2021. Two researchers who presented the study using a cover story recruited participants in person. Specifically, they explained that the study aimed to investigate how people perform simultaneous linguistic assignments and that they would be requested to perform at the same time multiple linguistic tasks. The cover story was necessary to disguise the real aim of the study to participants to avoid social desirability biases. Then, the researchers took notes of the participants’ willingness to accomplish the experimental task and scheduled an appointment with them at the Social Psychology lab.

**Figure 1:**
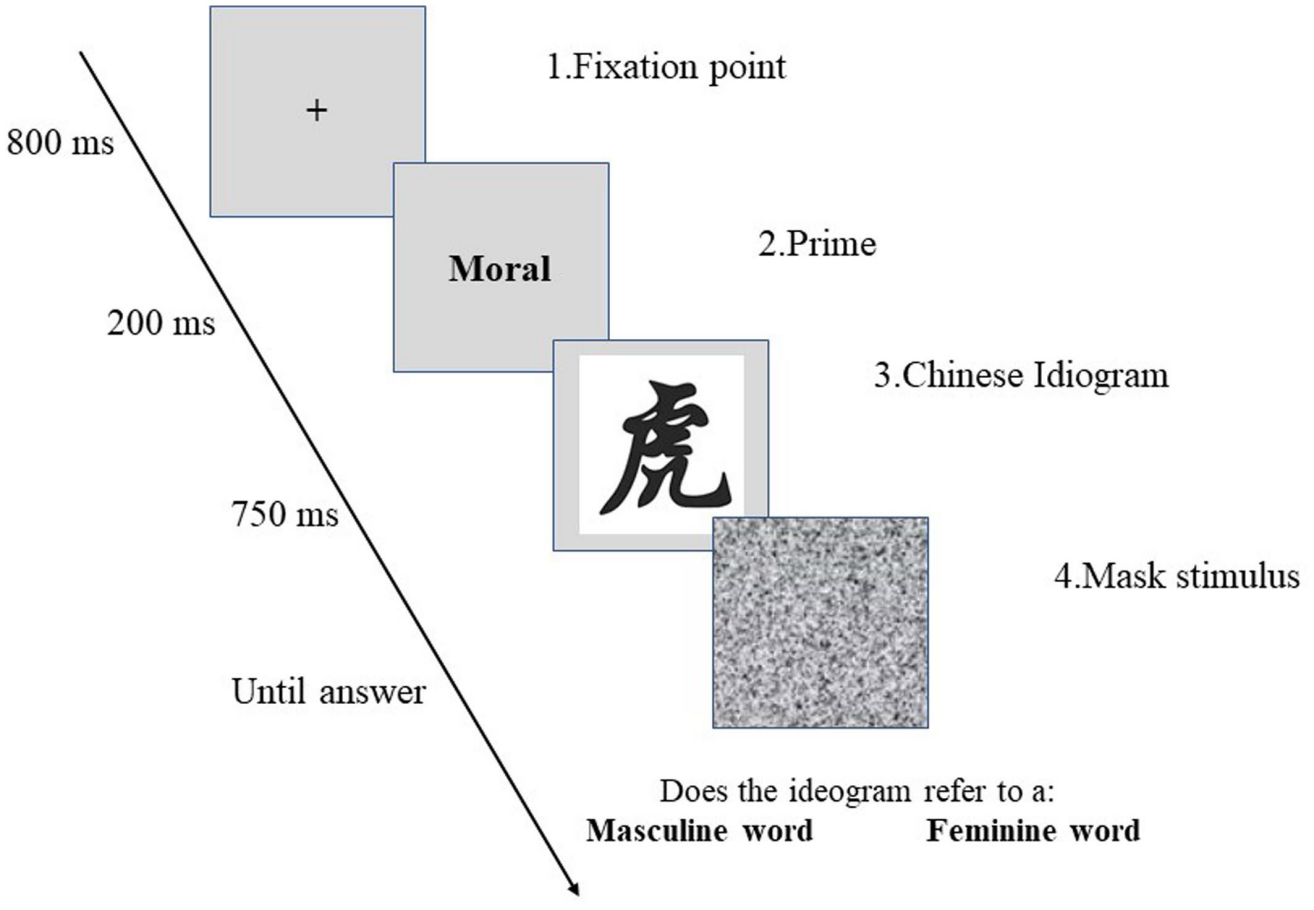
Illustration of a single trial in the adopted semantic misattribution procedure.

Once in the Laboratory, participants were seated in front of a computer screen and read the instructions concerning the tasks. They were told they would see pairs of stimuli shown below the other, the first being an English adjective and the second a Chinese ideogram. Participants were told that their task was to decide whether they thought that the ideogram represented a feminine or masculine word by pressing different buttons on the keyboard (i.e., A or L). Then, following the AMP procedure (Payne et al., 2005), participants were presented with a fixation point (800 ms) followed by a prime word (200 ms) and, after 135 ms, a Chinese ideogram (750 ms), as shown in Figure 1. The labels associated with the keyboards’ buttons varied among 12 blocks and were randomly chosen by the software (i.e., Inquisit Player) to avoid biases such as habituation and or bias due to the dominant hand of participants. Participants underwent the first trial with neutral English words (e.g., mirrored) to get familiar with the procedure. These evaluations were excluded from the analyses. The primes consisted of 15 words^1^ related to competence (i.e., competent, efficient, and intelligent), morality (i.e., sincere, honest, loyal), sociability (i.e., friendly, extraverted, sociable), dominance (i.e., competitive, ambitious, dominant), and Physical attractiveness (i.e., good looking, attractive, pretty). Every trait was randomly presented four times, equally distributed into 12 blocks for 60 trials. The time frame between blocks was 1,000 ms. We decided to administer prime adjectives in English to avoid bias due to the Italian language as a gendered language. In fact, in the Italian language, even adjectives are spelt differently based on the gender of the person or the object that it refers to, and no gender-neutral word exists. After the SMP task, participants filled in sociodemographic questions and were thanked and fully debriefed.

**Figure 2:**
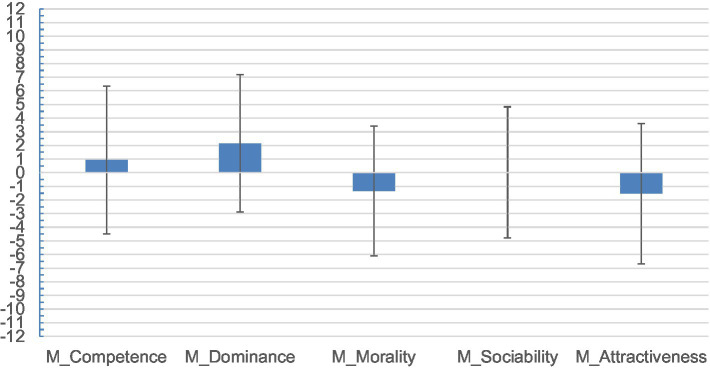
Associations of ideograms to the feminine and masculine domain as a function of primed dimension. Positive values indicate that ideograms are attributed to the masculine domain to a higher extent. Values close to 0 indicate equal attribution to the feminine and the masculine domain. Negative value indicates attributions to the feminine domain.

## 3. Results

Analyses were conducted using IBM SPSS software. After performing preliminary analysis (Table 1 reports correlations between study variables) we calculated two SMP scores obtained by summing feminine and masculine associations of each word used as priming trials, following the data analytic procedure used by Payne et al. (2005). Specifically, raw scores included four gendered attributions for each primed word, thus indicating whether the ideogram was considered to refer to a feminine or masculine word (e.g., Good looking: 1^st^ attribution = masculine, 2^nd^ attribution = feminine; 3^rd^ attribution = feminine; 4^th^ attribution = feminine. Pretty: 1^st^ attribution = feminine, 2^nd^ attribution = feminine; 3^rd^ attribution = feminine; 4^th^ attribution = feminine. Attractive: 1^st^ attribution = masculine, 2^nd^ attribution = masculine; 3^rd^ attribution = feminine; 4^th^ attribution = feminine). Then, we obtained two separate scores indicating the frequency with which each primed stimulus was attributed to a feminine or masculine ideogram, resulting in a number varying from 0 to 4 (e.g., good looking: *n* = 1 masculine; *n* = 3 feminine; pretty: *n* = 0 masculine; *n* = 4 feminine; attractive: *n* = 2 masculine; *n* = 2 feminine). Subsequently, we summed the frequencies with which the three words relating to each dimensions were attributed to either the masculine or the feminine domain (e.g., thus, overall, 9 attributions were for the feminine domain and 3 were to the masculine domain). Thus we obtained 10 scores five for competence, dominance, morality, sociability, and physical attractiveness, respectively, and five for the same dimensions related to the feminine domain.

**Table 1:** Descriptive statistics and correlations among the study variables.

Variable	*n*	*M*	*SD*	1	2	3	4	6
1. Competence	108	0.93	5.42	—				
2. Dominance	108	2.15	5.03	0.54^**^	—			
3. Morality	108	−1.35	4.76	0.34^**^	0.28^**^	—		
4. Sociability	108	0.28	4.80	0.34^**^	0.22^*^	0.23^*^	—	
6. Physical attractiveness	108	−1.54	5.13	−0.01	−0.22^*^	0.42^**^	0.18	—

*p < .05; **p < .01.

Then, we computed an overall comprehensive score by subtracting the overall masculine attribution score from the overall feminine score for each dimension. Scores of this variable could range from −12 to 12. Negative values indicated that, after the prime words, participants considered the ideographs to a greater extent as feminine, on the opposite positive values indicated that the ideographs were attributed to masculine domain. A zero value would imply that the corresponding dimension was equally attributed to males and females (Table 2).

**Table 2:** Means and Standard deviations of words attribution as a function of dimension.

Dimension			*t*(107)	*p*	Cohen’s d
	*M*	SD			
Competence	0.93	5.42	1.76	0.079	–
Dominance	2.15	5.03	4.44	0.001	0.45
Morality	−1.35	4.76	−2.95	0.004	0.40
Sociability	0.03	4.80	0.06	0.952	–
Physical attractiveness	−1.54	5.14	−3.11	0.002	0.43

### 3.1. Analysis of variance

We performed a repeated measure ANOVA^2^ with Dimension as a five-level within-participant factor and gender of participants as a between-participant factor to test whether the dimensions are attributed more to either the masculine or the feminine domain and to explore possible differences due to participants’ gender (Table 3). The analysis revealed a significant main effect of Dimension, due to the overall attribution of the different words to either masculine or feminine ideograms, *F*(4, 420) = 10.38, *p* < 0.001, *d* = 0.64. Pairwise comparisons using Bonferroni correction showed that being primed with Competence words led participants to consider ideograms as more masculine than when they were primed with Morality words, *p* < 0.001, 95% CI [1.02, 4.36] or Physical attractiveness words, *p* = 0.005, 95% CI [0.55, 4.90].

**Table 3:** Means and standard deviations of words attribution as a function of dimension and participants’ gender.

	Gendered attribution of traits					
	Male participants		Female participants	TOT		
	*M*	SD	*M*	SD	*M*	SD
Competence	2.28**	5.05	0.16	5.54	0.92	5.04
Morality	−1.55*	4.10	−1.38^*^	5.03	−1.44	4.70
Sociability	1.39	4.91	−0.72	4.64	0.03	4.82
Dominance	2.18**	4.87	2.10***	5.18	2.15	5.05
Physical Attractiveness	−1.26	5.36	−1.4*	5.06	−1.57	5.15

***p < 0.001; **p < 0.01; *p < 0.05.

Furthermore, after being exposed to Dominance primes, participants evaluated ideograms as more masculine than after having received Morality prime words, *p* < 0.001, 95% CI [1.92, 5.30], Sociability words, *p* = 0.040, 95% CI [0.05, 3.60], or Physical attractiveness, *p* < 0.001, 95% CI [1.35, 5.97]. Finally, ideograms were considered as more masculine after being primed with Sociability words than after being primed with Morality words, *p* = 0.028, 95% CI [−3.48, −0.12]. The Dimension × Gender of Participants’ interaction did not reach statistical significance, *F*(4, 420) = 2.07, *p* = 0.09.

### 3.2. *T*-test

However, since we were interested in exploring whether attributions elicited by different primes differed between female and male participants, we ran independent sample t-tests on each dimension. Only the *t*-test on sociability words was significant. After being exposed to Sociability words, male participants considered ideograms more masculine (*M* = 1.39; *SD* = 4.91) than female participants did (*M* = −0.72, *SD* = 4.64), *t*(105) = 2.26, *p* = 0.029, *d* = 0.44, 95% CI [0.22, 4.02]. Male participants when primed with competence words considered ideograms more masculine (*M* = 2.29, *SD* = 5.04) than female participants (*M* = 0.16, *SD* = 5.54), although the effect was not significant *t*(105) = 1.96, *p* = 0.052. No other significant results emerged in relation to the other dimensions. Findings showed that after being primed with Dominance words, both male (*M* = 2.18, *SD* = 4.88) and female (*M* = 2.13, *SD* = 5.18) participants evaluated the ideograms as masculine, *t(105)* = 0.52, *p* = 0.96. The opposite occurred for Morality and Physical attractiveness. Specifically, male participants (*M* = −1.55, *SD* = 4.10) and female participants (*M* = −1.38, *SD* = 5.03) chose the feminine option more after being primed with morality words, *t*(105), *p* = 0.85. Finally, both male (*M* = −1.26, *SD* = 5.36) and female participants (*M* = −1.74, *SD* = 5.06) evaluated as feminine the ideograms after being exposed to words referring to Physical Attractiveness, *t*(105) = 0.46, *p* = 0.65.

Finally, to assess the extent to which the single dimensions were overall attributed to either the masculine or the feminine domain separately for male and female participants, we conducted a series of one-sample *t*-tests against 0 (i.e., the mid-point of the femininity-masculinity score) as the fixed value of comparison. As reported in Table 3, male participants evaluated as masculine in the dimension of Competence, *t*(37) = 2.80, *p* = 0.008, *d* = 0.45, 95% CI [0.63, 3.95] and Dominance, *t*(37) = 2.76, *p* = 0.009, *d* = 0.45, 95% CI [0.58, 3.79]. Furthermore, male participants evaluated Sociability words as equally attributable to men and women, *t*(37) = 1.75, *p* = 0.09, 95% CI [−0.22, 3.01]. A similar non-significant result concerns males’ attribution of Physical Attractiveness primes, *t*(37) = −1.45, 95% CI [−1.26, −3.02]. Moreover, Morality words were considered by both male *t*(37) = −2.34, *p* = 0.025, *d* = 0.38, 95% CI [−2.90, −0.21] and female participants *t*(68) = −2.28, *p* = 0.026, *d* = 0.27 95% CI [−2.58, −0.17] as pertaining to the feminine domain. Female participants displayed associations to the masculine domain in relation to Dominance primes, *t*(68) = 3.14, *p* = 0.001, *d* = 0.41, 95% CI [0.89, 3.38] and associations to the feminine domain for Physical Attractiveness primes *t*(68) = −2.85, *p* = 0.006, *d* = 0.34, 95% CI [−2.96, −0.52]. Competence primes did not lead female participants to differentiate between the feminine and the masculine domains, *t*(68) = 0.24, *p* = 0.81, 95% CI [−1.17, 1.49]. Finally, female participants evaluated almost equally associated to both the feminine and masculine domain Sociability words, *t*(68) = −1.30, *p* = 0.20, 95% CI [−1.84, 0.39].”

## 4. Discussion

Extending and going beyond previous research, by adopting a multidimensional perspective (e.g., Prati et al., 2019; Pireddu et al., 2022), we tested whether Competence, Morality, Sociability, Dominance, and Physical Attractiveness were associated with either the feminine or the masculine domain at the implicit level. In this vein, the current study adopted an original approach by employing a Semantic Misattribution Procedure to examine gendered implicit beliefs along judgmental dimensions portraying women and men. The underlying idea of this work was that people spontaneously think of men when presented with specific traits (e.g., dominant) and women when primed with other traits (e.g., moral).

In general terms and in line with our hypotheses, results revealed that participants attributed higher masculinity to ideograms after being primed by Competence- or Dominance-related terms. In contrast, Morality and Physical attractiveness were attributed to feminine ideograms to a higher and significant extent than masculine ones. Surprisingly, Sociability was related to feminine and masculine ideograms almost to the same extent.

Furthermore, male participants perceived competence as more related to masculine ideograms, while female participants considered it to be equally related to feminine and masculine ideograms. Morality traits were perceived to be related to feminine ideograms to a higher extent than masculine ones by both male and female participants. As for Sociability traits, male participants considered it to be associated with masculine ideograms to a significant higher degree than feminine ones, whereas female participants attributed it to feminine ideograms to a little higher extent. Dominance was consensually attributed to masculine ideograms by male and female participants. Again, Physical Attractiveness was consensually attributed to feminine ideograms by both male and female participants. This is also in line recent findings, who showed that gender stereotypes have changed in such a way that contemporary gender stereotypes convey a substantial female advantage in communion and a smaller male advantage in the agency but also gender equality in competence along with some female advantage.

In this vein, findings revealed two profiles concerning the characteristics attributed to women and men domains, with two dimensions being common to both profiles, namely Competence, and Sociability. Thus, the masculine profile involves Competence, Dominance, and Sociability, while the feminine one implies Competence, Morality, Sociability, and Physical Attractiveness. Along this line, findings are consistent with the literature on male gender stereotypes by highlighting the presence of dominance as a stereotypical masculine dimension but also a novelty by revealing that men consider sociability as a masculine property to a high degree. Such a finding may reveal a slight change in how men consider themselves in the current time, where gender expectancies tend to become progressively more inter-gendered so that sociability characteristics are appreciated also by men as valuable traits. This is also in line with the evidence collected by Hentschel et al. (2019) on communality attributed to men and women. Following a different trend Kosakowska-Berezecka et al. (2023) found instead that gender gaps in communality are more pronounced in more egalitarian societies. These contrasting findings could be due to the specific measures employed in the different studies. Moreover, findings extended at the implicit level what has been already pointed out by the literature (e.g., Prati et al., 2019), namely, that women are evaluated along more dimensions than men given that competence, morality, sociability, and physical attractiveness were associated with feminine ideograms.

From a general point of view, findings support and extend quite consistently the literature (e.g., Moscatelli et al., 2020; Pireddu et al., 2022) by displaying competence and dominance as masculine characteristics to a higher extent, while morality and physical attractiveness were more consistently associated with the feminine domain (Menegatti et al., 2021). In general terms, the collected evidence shows that women are attributed traits primarily related to the capacity to build relationships (e.g., being honest and trustworthy). At the same time, men are usually considered to possess traits enabling them to be more goal-oriented, like being dominant (Williams and Tiedens, 2016). However, results went behind the literature, since men attributed to the masculine domain also sociability, which is usually associated with women. This finding can be interpreted as a change in male stereotypes recognizing sociability as a value that can also portray men. It should be noted, however, that these results were obtained from young men for whom sociability is important to be considered as popular guys among friends and mates. What has been found complements what Hentschel et al. (2019) revealed. In their work, men characterized themselves in less stereotypic terms, namely as more sociable (e.g., more friendly and extrovert). As we argue, the explanation provided by Hentschel et al. (2019) revolved around current changes in the perception of gender stereotypes.

Another sign of change resides in women associating competence with men and women almost equally. These findings likely stem from the work domain, where competence plays a crucial role and is required of women, even to a greater extent, as Biernat and Fuegen (2001) claimed. We can speculate that women are aware that in order to succeed, especially in the work domain, it is very important to be performative on the competence dimension. Competence is also one of the requirements that women are expected to display in the Perfection Bias literature reviewed above (Moscatelli et al., 2020). Moreover, as the literature on the Stereotype Content Model (Fiske et al., 2002) shows, competence is perceived as a high-status trait. It is thus very likely that women consider it as a means of enhancement of their status. In addition, the feminine domain was also associated with physical attractiveness. In this regard, our evidence is consistent with the work of Ramati-Ziber et al. (2020) concerning beauty expectancies. They argue that those beliefs represent social standards in our society and not pursuing principles of beauty can bring a backlash effect on women. In other words, prescriptive beauty norms determine socially desirable characteristics for women (e.g., using makeup, high heels, perfect skin), which are associated with their traditional lower power role and rewards (e.g., being sexually desirable; access to greater resources). Furthermore, the phenomenon that seems to emerge is that women themselves displayed the associations between physical attractiveness and the feminine domain, implying that they may have likely internalized the expectancies related to their physical appearance since, this is related to several positive aspects such as perceived higher status and more popularity (e.g., Fisher et al., 2019). Along this line, they can incur negative consequences when these expectancies are not met. Moreover, in general terms, the feminine domain is composed of four dimensions while the masculine one only by three dimensions rendering women’s expected standard more difficult to be achieved, especially if they are not physically attractive. What is very important for this contribution is that the multidimensional associations to the female domain point to a “perfection bias” toward women at the implicit level that can render it even more difficult for them to meet the required multiple expectancies.

### 4.1. Strengths, limitations, and future directions

The present study should be considered also for its strengths and shortcomings, which suggest directions for future research. This study highlights the implicit semantic associations concerning the main social judgment dimensions. Although the Semantic Misattribution Procedure displays good statistical indices (e.g., Ye and Gawronski, 2018), the effects obtained with one measure may not generalize to other measures to the extent that these effects are driven by method-related processes (e.g., Gawronski et al., 2008). This is because performance on various measures can be driven by various processes. Therefore, it is advisable to replicate these findings using alternative measures to ensure appropriate interpretations of the results obtained with a specific measure (Gawronski et al., 2008). Furthermore, the current study includes five dimensions (i.e., Competence, Dominance, Morality, Sociability, and Physical Attractiveness), each constituted by three characteristics (i.e., our prime words). However, the social judgment dimensions have been defined through several characteristics (e.g., Hentschel et al., 2019; Menegatti et al., 2021; Pireddu et al., 2022). Therefore, future research could enlarge the prime words to deepen the understanding of these processes, even from an implicit point of view. Finally, even if our main focus did not consist in investigating gender differences, the explorative analysis that we conducted unveiled interesting aspects related to the differences made by male and female participants. Therefore, future research may want to consider it as an integral part of the experimental design and, consequently, reach an equal representation of the sample.

To conclude, findings might pave the way to further investigation of the expectations embedded in social judgments and provide a means of raising awareness of the implicit processes that mainly influence women in several spheres of life. It is possible, for instance, that gendered beliefs have a stronger correlation with hiring decisions, performance reviews, or pay scales. It would be helpful to analyze whether and how these perceptions change depending on the situation in which they are activated to understand the social judgment dimensions better.

### 4.2. Conclusion

This study highlights how semantic associations between social judgment dimension and masculine and feminine representations are activated implicitly. We can point out some intriguing results. On the one hand, results concerning attributions from primes related to competence and sociability suggest valuable novelty, namely the perceptions of social judgment dimensions and the stereotypical way they are expressed are undergoing changes. On the other hand, results underline how deeply these representations are embedded in our culture. The associations related to physical attractiveness can be taken as an example. In this case, female participants expressed stronger associations between this dimension and the feminine domain. These findings have interesting implications for practitioners. For instance, training on gender bias in organizations, schools, or other contexts might employ the SMP procedure in programs aimed at raising individuals’ awareness of their stereotypical beliefs and their pervasive effects. Overall, the results provided a complex picture that reveals that multiple characteristics are used to define (expectancies toward) women and men and highlights how they may be interiorized and are evolving along a multifaceted structure even stronger with reference to women.

## Data availability statement

The datasets presented in this study can be found in online repositories. The names of the repository/repositories and accession number (s) can be found at: https://osf.io/znr6b/.

## Ethics statement

The study involving humans was approved by the Bioethical Committee of the University of Bologna (Prot. n. 314865). The studies were conducted in accordance with the local legislation and institutional requirements. The participants provided their written informed consent to participate in this study.

## Author contributions

SP: Data curation, Formal analysis, Investigation, Project administration, Writing – original draft, Writing – review & editing. MR: Conceptualization, Methodology, Project administration, Supervision, Validation, Writing – original draft, Writing – review & editing. VG: Conceptualization, Investigation, Methodology, Project administration, Software, Writing – original draft. MM: Conceptualization, Methodology, Validation, Writing – review & editing. SM: Conceptualization, Methodology, Project administration, Supervision, Validation, Writing – review & editing.

## Footnotes

^1^ The prime words related to Competence, Morality, Dominance and Sociability were drawn from Pireddu et al. (2022), while Physical Attractiveness characteristics were taken from Menegatti et al. (2021).

^2^Figure 2 shows a graphical representation of the results.
